# Association of the serum calcium level with metabolic syndrome and its components among adults in Taiwan

**DOI:** 10.20945/2359-3997000000632

**Published:** 2023-05-29

**Authors:** Jer-min Chen, Tai-yin Wu, Yi-fan Wu, Kuan-liang Kuo

**Affiliations:** 1 Taipei City Hospital Renai Branch Department of Family Medicine Taipei Taiwan Department of Family Medicine, Renai Branch, Taipei City Hospital, Taipei, Taiwan; 2 University of Taipei Department of Psychology and Counseling Taipei Taiwan Department of Psychology and Counseling, University of Taipei, Taipei, Taiwan; 3 Taipei City Hospital Zhongxing Branch Department of Family Medicine Taipei Taiwan Department of Family Medicine, Zhongxing Branch, Taipei City Hospital, Taipei, Taiwan; 4 National Taiwan University Institute of Epidemiology and Preventive Medicine Taipei Taiwan Institute of Epidemiology and Preventive Medicine, National Taiwan University, Taipei, Taiwan; 5 National Chengchi University Department of Psychology Taipei Taiwan Department of Psychology, National Chengchi University, Taipei, Taiwan; 6 National Yang Ming Chiao Tung University Institute of BioMedical Informatics Taipei Taiwan Institute of BioMedical Informatics, National Yang Ming Chiao Tung University, Taipei, Taiwan

**Keywords:** Metabolic syndrome, serum calcium, hypertension, hyperglycemia, obesity

## Abstract

**Objective:**

An increasing amount of literature indicates that the serum calcium level may be related to metabolic syndrome (MetS) and obesity. This study aimed to examine the relationship between the serum calcium level and MetS in adults in Taiwan.

**Subjects and methods:**

We conducted a cross-sectional study and enrolled 1,580 participants (54.4% women; mean age, 33.28 ± 12.21 years) who underwent health examinations in northern Taiwan between 2012 and 2016. Logistic regression was performed to estimate the odds ratios (ORs) and 95% confidence intervals (CIs) for the risk of MetS and its components in groups of patients in the tertiles of the serum calcium level.

**Results:**

In total, 167 participants (10.6%) had MetS. The odds of high systolic blood pressure (BP), blood glucose, and triglyceride (TG) levels significantly increased as the serum calcium level increased. Compared with the participants in the lowest tertile of the serum calcium level (tertile 1), those in the second tertile (OR = 1.47, 95% CI: 0.97-2.23) and third tertile (OR = 1.63, 95% CI: 1.06-2.53) had a significantly higher risk of MetS. Further analyses revealed a significant association between MetS and an increased serum calcium level in those in the overweight and obese groups. However, there was no association between the serum calcium levels and MetS in those in the normal weight group.

**Conclusion:**

This study demonstrated that a higher serum calcium level is associated with an increased risk of MetS and its components in adults with overweight and obesity.

## INTRODUCTION

Metabolic syndrome (MetS) is a combination of glucose intolerance, central obesity, a high triglyceride (TG) level, a low high-density lipoprotein cholesterol (HDL-C) level, and hypertension. The prevalence rates of MetS and its components are increasing worldwide (1), even in young adults (2). MetS predicts the onset of metabolic diseases, such as type 2 diabetes mellitus and cardiovascular diseases (3,4). Moreover, MetS is linked to cardiovascular mortality and all-cause mortality and represents high utilization and expenses in patients receiving medical care (5).

The serum calcium level plays an important role in glucose homeostasis (6), and it enhances the vascular resistance (7). Previous epidemiological studies have demonstrated that high serum calcium levels are associated with high blood pressure (BP) levels (8), high glucose levels (9), and dyslipidemia (10). However, other literature has reported inverse associations between the serum calcium level and the individual components of MetS (11,12). Research suggests that an increased serum calcium level is associated with an increased risk of MetS (13). However, Baek and cols. reported that there was no positive correlation between the serum calcium level and the incident risk of MetS (14). Previous studies have reported inconsistent results on the association between obesity and serum calcium level with some reporting a positive correlation between the two, while others an inverse correlation (15,16). Additionally, limited studies have investigated the effect of obesity on the serum calcium level and MetS. Furthermore, most previous studies designed to determine the effects of serum calcium on MetS were conducted in older participants (17). Studies determining an association between the serum calcium level and MetS in young adults are scarce.

Therefore, this cross-sectional study investigated the association between the serum calcium level and MetS in young adults in Taiwan.

## SUBJECTS AND METHODS

This study retrospectively examined the medical records of 1,688 adults who participated in health examinations at Taipei City Hospital in northern Taiwan from January 2012 to December 2016. We included participants aged 18 years and older in the analysis. We excluded participants based on the following criteria: participants with clearance rate less than 30 mL/min according to the Cockgroft–Gault formula; participants whose lipid profile data were unavailable; or had TG level > 400 mg/dL. Participants were asked to complete a self-administered questionnaire that included questions on lifestyle habits (average daily alcohol intake and cigarette smoking), medical illness, and current medications. The study protocol was approved by the institutional review board of Taipei City Hospital (#TCHIRB-10604113-E).

Height and weight were measured using a stadiometer and digital scale (HW-868, Super-View Medical, Taoyuan, Taiwan). The participants were asked to wear light clothes without shoes. Height was measured to the nearest 0.1 cm, and weight was measured to the nearest 0.1 kg. Body mass index (BMI) was calculated as the weight (kg) divided by the height in meters squared (m^2^). Waist circumference was measured midway between the lowest ribs and iliac crest in a horizontal plane in a standing position. BP was measured in the right arm with at least 10 min of rest in a sitting position using a validated automated oscillometric device (Easy X 800R, Jawon, Seoul, South Korea).

All blood samples were collected in the morning after an overnight fasting. The serum glucose, total cholesterol, TG, and low-density lipoprotein cholesterol (LDL-C) levels were determined using an autoanalyzer (Cobas c 702; Roche, Basel, Switzerland). The serum HDL-C concentration was calculated using Friedewald's formula (18). The normal serum calcium levels ranged from 8.1 to 10.4 mg/dL. The study participants were categorized into tertiles according to their serum calcium levels. The serum calcium levels were categorized into tertiles (<9.3, 9.3-9.6, and ≥9.6 mg/dL in men; and <9.1, 9.1-9.4, ≥9.4 mg/dL in women).

To define MetS, we adopted the 2005 revision of the National Cholesterol Education Program (NCEP) Adult Treatment Panel III (ATP III) (19). The criteria for abdominal obesity were modified according to the 2000 WHO Asia Pacific Guidelines (20), which defined obesity as a waist circumference of ≥90 cm and ≥80 cm for men and women, respectively. Participants with at least three of the following five components were classified as having MetS: (1) systolic BP ≥ 130 mmHg, diastolic BP ≥ 85 mmHg, or use of BP-lowering medication; (2) TG ≥ 150 mg/dL or use of medication for an elevated TG level; (3) serum glucose ≥ 100 mg/dL or use of antidiabetic medication; (4) HDL-C < 40 mg/dL for men and < 50 mg/dL for women or use of medication for a reduced HDL; and (5) waist circumference ≥ 90 cm for men and ≥ 80 cm for women. The definitions of obesity (BMI ≥ 30 kg/m^2^) and overweight (BMI 25 to <30 kg/m^2^) by WHO are based mainly on criteria derived from studies involving subjects of European origins and the BMI cut-off point (≥30 kg/m^2^) might be too high for Asians, thereby underestimating associated health risks (21). Therefore, we defined obesity (BMI ≥ 27 kg/m^2^) and overweight (BMI: 24 to <27 kg/m^2^) using the definition of the Ministry of health and Welfare of Taiwan (22). Normal weight is defined as 18.5 to <24 kg/m^2^ in Taiwan.

The data are presented as the mean ± SD for continuous variables and as the number or percentage for categorical variables. The normal distribution of all continuous variables was determined using the Kolmogorov–Smirnov test. If necessary, logarithmic transformation was performed to achieve a normal distribution. The clinical and biochemical characteristics were compared using the chi-square test or analysis of variance when the variables were categorical or continuous, respectively. Multiple logistic regression analysis was performed to calculate the odds ratios (ORs) and confidence intervals (CIs) of MetS and its components. p<0.05 was considered statistically significant. All data were analyzed using SAS for Windows, version 9.4, statistical software (SAS Institute Inc., Cary, NC, USA).

## RESULTS

A total of 1,580 participants (859 women and 721 men) with a mean age of 33.28 ± 12.21 years were enrolled and more than 80% adults younger than 40 years. The levels of serum calcium were 9.28 ± 0.34 mg/dL in women and 9.49 ± 0.28 mg/dL in men. Sixty women (7.0%) and 107 men (14.8%) had MetS. The anthropometric and biochemical characteristics according to tertiles of serum calcium levels are presented in Table 1. The distribution of sex was equal among the three groups, and participants in the higher tertiles were more likely to be young and have a lower body mass index. The total cholesterol, HDL-C, and LDL-C levels increased linearly in participants with the lowest to the highest serum calcium tertiles. The prevalence rates of daily smoking and alcohol intake in our study population was 11.8% and 1.2%, respectively.

**Table 1:** Basic characteristics of the study population

		Serum calcium tertile			All	p value
		Tertile 1	Tertile 2	Tertile 3		
Numbers		553	550	477	1580	
Age (yr)		35.60 ± 13.37	32.64 ± 11.93	31.34 ± 10.59	33.28 ±12.21	<0.0001
	<30	303	365	340	1008	
	30-40	113	97	77	287	
	>40	137	88	60	285	
Serum calcium (mg/dL)		9.02 ± 0.21	9.39 ± 0.12	9.77 ± 0.24	9.38 ± 0.36	<0.0001
Female gender (%)		56.24	53.45	53.25	54.37	0.56
Height (cm)		165.23 ± 8.80	166.40 ± 8.81	166.17 ± 9.04	165.92 ± 8.89	0.06
Weight (kg)		64.23 ± 14.68	64.43 ± 14.72	63.38 ± 15.12	64.04 ± 14.82	0.31
BMI (kg/m^2^)		23.39 ± 4.27	23.14 ± 4.29	22.78 ± 4.26	23.12 ± 4.28	0.02
WC (cm)		78.49 ± 11.85	77.64 ± 11.97	76.77 ± 12.33	77.67 ± 12.05	0.03
Systolic BP (mmHg)		112.13 ± 15.40	113.49 ± 15.48	113.67 ± 14.60	113.08 ± 15.20	0.10
Diastolic BP (mmHg)		67.24 ± 10.13	68.27 ± 10.46	68.27 ± 9.37	67.91 ± 10.03	0.17
FBG (mg/dL)		88.08 ± 12.99	88.91 ± 15.62	89.63 ± 19.40	88.83 ± 16.05	0.61
TC (mg/dL)		179.62 ± 32.39	184.02 ± 33.06	191.04 ± 31.77	184.60 ± 32.75	<0.0001
HDL-C (mg/dL)		60.68 ± 17.66	61.99 ± 18.19	63.53 ± 13.23	62.00 ± 18.35	0.03
LDL-C (mg/dL)		101.39 ± 28.02	104.49 ± 30.87	108.86 ± 29.19	104.72 ± 29.52	0.001
TG (mg/dL)		89.76 ± 53.32	90.19 ± 51.27	94.89 ± 59.82	91.46 ± 54.70	0.45
Daily smoking (%)		11.03	11.09	13.63	11.82	0.35
Daily alcohol intake (%)		1.27	1.45	0.84	1.21	0.66

BMI: body mass index; WC: waist circumference; BP: blood pressure; FBG: fasting blood glucose; TC: total cholesterol; HDL-C: high-density lipoprotein cholesterol; LDL-C: low-density lipoprotein cholesterol; TG: triglycerides.

Tertile 1: females: 8.1 mg/dL ≤ serum calcium < 9.1 mg/dL, males: 8.1 mg/dL ≤ serum calcium < 9.3 mg/dL; tertile 2: females: 9.1 mg/dL ≤ serum calcium < 9.4 mg/dL, males: 9.3 mg/dL ≤ serum calcium < 9.6 mg/dL; tertile 3: females: 9.4 mg/dL ≤ serum calcium < 10.4 mg/dL, males: 9.6 mg/dL ≤ serum calcium < 10.4 mg/dL.

Analysis of variance was performed to compare the differences between groups for the continuous variables, and the chi-square test was performed to compare the differences between groups for the categorical variables.

Plus-minus values are presented as the mean ± SD.

Pearson's coefficients of correlation between serum calcium levels and the components of MetS are shown in Supplement 1. Positive but weak correlations were observed between the serum calcium level and waist circumference, systolic BP, diastolic BP, serum glucose level, total cholesterol level, LDL-C level, and TG level.

Table 2 shows the association between serum calcium levels and MetS and its components. The serum calcium level was positively associated with the risk of MetS (OR = 2.28, 95% CI: 1.42-3.69). After further adjustment for age, sex, cigarette smoking, and alcohol intake, this association remained significant. Regarding the components of MetS, the serum calcium level was also associated with the risk of high systolic BP, high blood glucose level, and high TG level. The serum calcium level was not significantly associated with abdominal obesity or a low HDL-C level.

**Table 2:** Logistic regression analysis of metabolic syndrome and its individual components with the serum calcium level

		Crude	Model 1^a^	Model 2^b^
		OR (95% CI)	OR (95% CI)	OR (95% CI)
Metabolic syndrome		1.87 (1.21-2.89)	2.31 (1.43-3.83)	2.28 (1.42-3.69)
Abdominal obesity		0.82 (0.59-1.13)	0.79 (0.56-1.14)	0.80 (0.56-1.14)
High BP		1.28 (0.89-2.84)	1.30 (0.86-1.97)	1.30 (0.86-1.97)
	High systolic BP	1.54 (1.04-2.27)	1.52 (0.99-2.34)	1.51 (0.99-2.33)
	High diastolic BP	1.32 (0.72-2.31)	1.09 (0.60-2.00)	1.12 (0.61-2.04)
High glucose		1.09 (0.68-1.74)	1.83 (1.06-3.15)	1.82 (1.06-3.14)
Low HDL-C		1.15 (0.81-1.64)	1.16 (0.79-1.70)	1.15 (0.78-1.68)
High TG		1.95 (1.31-2.91)	2.12 (1.37-3.28)	2.13 (1.38-3.29)

OR: odds ratio; CI: confidence interval; BP: blood pressure; HDL-C: high-density lipoprotein cholesterol; TG: triglycerides.

Abdominal obesity (waist circumference ≥ 80 cm in women; waist circumference ≥ 90 cm in men), high blood pressure (BP) (systolic/diastolic BP ≥ 130/85 mmHg or use of BP-lowering medication), high triglyceride (TG) level (TG level ≥ 150 mg/dL or use of medication for an elevated TG level), low high-density lipoprotein-cholesterol (HDL-C) level (HDL-C < 50 mg/dL in women or HDL-C < 40 mg/dL in men; or use of medication for a reduced HDL-C), and high glucose level (glucose level ≥ 100 mg/dL or use of antidiabetic medication).

a adjusted for age and gender.

b adjusted for age, gender, smoking, and alcohol intake.

The relationships between the serum calcium level and MetS was analyzed using logistic regression (Table 3). We observed that participants with a serum calcium level in the second and third tertiles had a 1.47-fold and 1.63-fold increased MetS risk, respectively, compared to those with levels in the first tertile (p for trend = 0.03) after adjusting for confounding factors.

**Table 3:** Multivariate analysis of the association between serum calcium and MetS

	Serum calcium, mg/dL			p for trend
	Tertile 1	Tertile 2	Tertile 3	
Numbers	553	550	477	
Crude (95% CI)	1 (reference)	1.15 (0.78-1.70)	1.13 (0.76-1.69)	0.54
Model 1^a^ (95% CI)	1 (reference)	1.48 (0.98-2.25)	1.66 (1.08-2.57)	0.02
Model 2^b^ (95% CI)	1 (reference)	1.47 (0.97-2.23)	1.63 (1.06-2.53)	0.03

MetS, metabolic syndrome; CI, confidence interval.

a adjusted for age and sex.

b adjusted for age, gender, smoking, and alcohol intake.

In further analyses, we observed that an increased serum calcium level was significantly associated with the risk of MetS for those in the second tertile (OR = 1.74, 95% CI: 1.06-2.84) and third tertile (OR = 2.15, 95% CI: 1.28–3.61) who were in the overweight and obesity groups. The association between the serum calcium level and MetS in the normal-weight participants was not significant (Table 4). Six participants had a serum calcium level outside of the normal range. Restricting the analysis to the participants with a normal calcium level did not alter these findings (Supplement 2).

**Table 4:** Multivariate analysis of the association between serum calcium and MetS stratified by BMI

		Serum calcium			p for trend
		Tertile 1	Tertile 2	Tertile 3	
Overweight/obesity^a^					
	Numbers	201	190	148	
	Crude	1 (reference)	1.43 (0.90-2.27)	1.65 (1.02-2.70)	0.04
	Model 1^c^ (95% CI)	1 (reference)	1.73 (1.06-2.83)	2.15 (1.28-3.61)	0.003
	Model 2^d^ (95% CI)	1 (reference)	1.74 (1.06-2.84)	2.15 (1.28-3.61)	0.003
Normal weight^b^					
	Numbers	328	325	294	
	Crude	1 (reference)	0.75 (0.31-1.80)	0.65 (0.25-1.66)	0.10
	Model 1 (95% CI)	1 (reference)	1.00 (0.38-2.62)	1.26 (0.44-3.58)	0.69
	Model 2 (95% CI)	1 (reference)	0.87 (0.33-2.31)	1.14 (0.40-3.25)	0.86

MetS, metabolic syndrome; BMI, body mass index; CI, confidence interval.

a Overweight/obesity: BMI ≥ 24 kg/m^2^.

b Normal weight: 18.5 kg/m^2^ ≤ BMI < 24 kg/m^2^.

c Adjusted for age and sex.

d Adjusted for age, gender, smoking, and alcohol intake.

## DISCUSSION

The current study demonstrated that a higher serum calcium level was associated with higher levels of systolic BP, blood glucose, and TG. Our findings suggest that the higher the calcium level, the greater the risk of MetS in overweight and obese young adults. However, no significant association was observed between the serum calcium level and MetS in the normal-weight participants.

More than 10% of the adults in our study had MetS. The prevalence of MetS in our adult population was higher than that in a nationally representative sample from Taiwan investigated in 2002 (23). Similarly, Chuang and cols. reported a prevalence rate of MetS of 6.1% in a young adult population who underwent health examinations in 2000 (24). The increasing trend in the prevalence of MetS is consistent with the findings of a previous study (2).

We reported lower serum calcium levels in women than in men, which is a finding that is in agreement with previous evidence (25). However, Koek and cols. suggested that women had a higher serum calcium level than men in participants aged > 45 years and that there were age-dependent sex differences in the serum calcium level (26). Further studies are needed to determine whether hormonal fluctuations influence the serum calcium level in different age and sex groups.

Aoki and Miyagawa discovered that an increased serum calcium level has been shown to cause a calcium influx into the arterial muscle, which induces vasoconstriction, resulting in an elevated blood pressure and increased peripheral vascular resistance (27). The results of studies on the association between the serum calcium level and BP have been conflicting. Our study reported that the serum calcium level was positively associated with the risk of a high systolic BP, which is consistent with the findings of previous research. Sun and cols. demonstrated that the risk of hypertension and high systolic BP in adolescents with a higher serum calcium level was 1.89-times and 2.02-times higher than in those with lower levels (8). Sabanayagam and cols., in a large cross-sectional study of 12,405 participants in the United States, reported that the total serum calcium level was positively associated with hypertension (28). However, Cho and cols. reported that the serum calcium level was not associated with an increased risk of high BP (13). A possible explanation for this difference may be partly due to the exclusion of participants taking medications for hypertension in that study. Furthermore, Kunutsor and Laukkanen demonstrated an inverse association between the serum ionized calcium level and hypertension in middle-aged men in Finland (11). The discrepancy of correlation may due to the reason that the association between the serum ionized calcium level and BP may vary by the plasma renin level (29). Hunt and cols. demonstrated a positive association between the ionized calcium level and BP in the high-renin group and an inverse association in the low renin level group.

Altered serum calcium concentrations could lead to pancreatic β-cell dysfunction, which could subsequently contribute to impaired insulin secretion (30). Hagström and cols. found that serum calcium levels were independently associated with insulin sensitivity in a community cohort (31). We demonstrated that serum calcium levels were positively associated with glucose levels and the risk of high glucose levels. These findings are in line with the results of previous research on middle-aged and elderly individuals (13).

The potential mechanisms involved in the relationship between serum calcium levels and waist circumference are unclear. Ahlström and cols. discovered a positive correlation between the serum calcium level and waist circumference in an elderly population (32). In this study, serum calcium levels were not associated with waist circumference. Similarly, a study of 1,137 Korean adults found no significant association between serum calcium levels and central obesity (33).

Previous reports have suggested that serum calcium can decrease cholesterol catabolism in the liver and stimulate lipid synthesis (34). Cho and cols. suggested that TG levels increase with high serum calcium levels (13). Our findings also showed that serum calcium levels were positively associated with the risk of high TG levels. In this study, the serum calcium levels were not significantly associated with low HDL-C levels. Kim and cols. discovered that serum calcium was positively associated with low HDL-C levels in middle-aged and elderly population (33). The discrepancy may be partly explained by sex and age group of study participants, and the literature has indicated that calcium supplementation might increase serum cholesterol by decreasing hepatic catabolism in estrogen deficiency conditions (35).

In the present study, we found a positive association between serum calcium levels and the risk of MetS, which is in agreement with previous evidence (33). MetS and obesity are associated with chronic low-grade inflammation, in which there are elevated pro-inflammatory cytokines. Cifuentes and cols. indicated that obesity-related pro-inflammatory cytokines increase calcium-sensing receptor (CaSR) protein expression in human adipocytes and that CaSR plays a vital role in regulating calcium homeostasis (36). However, Vaidya and cols. reported that calcium regulatory hormones may have inconsistent effects on adipocytokines or inflammatory markers in obese and lean healthy participants (37). Therefore, we further analyzed the effect of obesity on serum calcium levels and MetS using BMI stratification. We found that the highest tertile was associated with a higher risk of MetS than the lowest tertile in obese and overweight participants. This association was not observed in normal-weight participants. However, Baek and cols. discovered a decreased risk of incident MetS associated with increasing serum calcium levels in participants with central obesity at baseline (14). This contradiction may partly be related to the selection of participants with similar metabolic risk factors. Further studies elucidating the mechanism of the modification effect of obesity on the risk of serum calcium levels and MetS are warranted.

Our study has some limitations. First, because of the cross-sectional design of this study, we could not verify causal relationships between serum calcium levels and MetS and its components. Second, we performed investigation with total serum calcium level, which might be affected by serum albumin and PH. Third, parathyroid hormone (PTH) and vitamin D levels were known to be associated with MetS (32,38). Under the restriction of analysis in normocalcemic participants, our results suggest that the association is not influenced by individuals with abnormal levels of serum calcium, such as hyperparathyroidism or malignancy. While Kim and cols. found that the association between serum calcium and MetS was unchanged after adjusting for vitamin D and PTH (33), this would have been a complete study if the two elements were collected for analysis.

In summary, we report an association between high serum calcium levels and a higher risk of MetS and its components in Taiwan, especially in obese participants. Further prospective research is necessary to fully determine the association between serum calcium levels and risk of developing MetS in young adults.

## Supplement 1 Correlation^a^ between the serum calcium level and the individual components of metabolic syndrome

**Table:**

	r	p value
Age	-0.185	<0.001
BMI	0.041	0.106
WC	0.079	0.002
Systolic BP	0.146	<0.001
Diastolic BP	0.130	<0.001
Glucose	0.096	0.001
TC	0.135	<0.001
HDL-C	-0.084	0.001
LDL-C	0.150	<0.001
TG	0.111	<0.001

BMI: body mass index; WC: waist circumference; BP: blood pressure; TC: total cholesterol; HDL-C: high-density lipoprotein cholesterol; LDL-C: low-density lipoprotein cholesterol; TG: triglycerides.

a Pearson's coefficients.

## Supplement 2 Multivariate analysis of the association between a serum calcium level within the normal range and MetS stratified by BMI

**Table:**

		Serum calcium			p for trend
		Tertile 1	Tertile 2	Tertile 3	
Overweight/obesity^a^					
	Numbers	201	190	145	
	Model 1^c^ (95% CI)	1 (reference)	1.73 (1.06-2.83)	2.08 (1.23-3.51)	0.005
	Model 2^d^ (95% CI)	1 (reference)	1.73 (1.06-2.83)	2.08 (1.24-3.51)	0.005
Normal weight^b^					
	Numbers	327	325	292	
	Model 1 (95% CI)	1 (reference)	1.00 (0.38-2.61)	1.25 (0.44-3.58)	0.69
	Model 2 (95% CI)	1 (reference)	0.87 (0.33-2.30)	1.14 (0.40-3.25)	0.86

MetS, metabolic syndrome; BMI, body mass index; CI, confidence interval.

a Overweight/obesity: body mass index ≥ 24 kg/m^2^.

b Normal weight: 18.5 kg/m^2^ ≤ body mass index < 24 kg/m^2^.

c Adjusted for age and gender.

d Adjusted for age, gender, smoking, and alcohol intake.

## References

[1] Nolan PB, Carrick-Ranson G, Stinear JW, Reading SA, Dalleck LC (2017). Prevalence of metabolic syndrome and metabolic syndrome components in young adults: A pooled analysis. Prev Med Rep.

[2] Mattsson N, Rönnemaa T, Juonala M, Viikari JS, Raitakari OT (2007). The prevalence of the metabolic syndrome in young adults. The Cardiovascular Risk in Young Finns Study. J Intern Med.

[3] Lakka HM, Laaksonen DE, Lakka TA, Niskanen LK, Kumpusalo E, Tuomilehto J (2002). The metabolic syndrome and total and cardiovascular disease mortality in middle-aged men. JAMA.

[4] Ninomiya JK, L'Italien G, Criqui MH, Whyte JL, Gamst A, Chen RS (2004). Association of the metabolic syndrome with history of myocardial infarction and stroke in the Third National Health and Nutrition Examination Survey. Circulation.

[5] Boudreau DM, Malone DC, Raebel MA, Fishman PA, Nichols GA, Feldstein AC (2009). Health care utilization and costs by metabolic syndrome risk factors. Metab Syndr Relat Disord.

[6] Becerra-Tomás N, Estruch R, Bulló M, Casas R, Díaz-López A, Basora J (2014). Increased serum calcium levels and risk of type 2 diabetes in individuals at high cardiovascular risk. Diabetes Care.

[7] Hazari MA, Arifuddin MS, Muzzakar S, Reddy VD (2012). Serum calcium level in hypertension. N Am J Med Sci.

[8] Sun H, Shi J, Wang H, Fu L, Zhou B, Wu X (2013). Association of serum calcium and hypertension among adolescents aged 12-17 years in the rural area of Northeast China. Biol Trace Elem Res.

[9] Yamaguchi T, Kanazawa I, Takaoka S, Sugimoto T (2011). Serum calcium is positively correlated with fasting plasma glucose and insulin resistance, independent of parathyroid hormone, in male patients with type 2 diabetes mellitus. Metabolism.

[10] Gallo L, Faniello MC, Canino G, Tripolino C, Gnasso A, Cuda G (2016). Serum Calcium Increase Correlates With Worsening of Lipid Profile: An Observational Study on a Large Cohort From South Italy. Medicine (Baltimore).

[11] Kunutsor SK, Laukkanen JA (2017). Circulating active serum calcium reduces the risk of hypertension. Eur J Prev Cardiol.

[12] Kim KN, Oh SY, Hong YC (2018). Associations of serum calcium levels and dietary calcium intake with incident type 2 diabetes over 10 years: the Korean Genome and Epidemiology Study (KoGES). Diabetol Metab Syndr.

[13] Cho GJ, Shin JH, Yi KW, Park HT, Kim T, Hur JY (2011). Serum calcium level is associated with metabolic syndrome in elderly women. Maturitas.

[14] Baek JH, Jin SM, Bae JC, Jee JH, Yu TY, Kim SK (2017). Serum Calcium and the Risk of Incident Metabolic Syndrome: A 4.3-Year Retrospective Longitudinal Study. Diabetes Metab J.

[15] Ren XH, Yao YS, He LP, Jin YL, Chang WW, Li J (2013). Overweight and obesity associated with increased total serum calcium level: comparison of cross-sectional data in the health screening for teaching faculty. Biol Trace Elem Res.

[16] Zohal M, Jam-Ashkezari S, Namiranian N, Moosavi A, Ghadiri-Anari A (2019). Association between selected trace elements and body mass index and waist circumference: A cross sectional study. Diabetes Metab Syndr.

[17] Saltevo J, Niskanen L, Kautiainen H, Teittinen J, Oksa H, Korpi-Hyövälti E (2011). Serum calcium level is associated with metabolic syndrome in the general population: FIN-D2D study. Eur J Endocrinol.

[18] Friedewald WT, Levy RI, Fredrickson DS (1972). Estimation of the concentration of low-density lipoprotein cholesterol in plasma, without use of the preparative ultracentrifuge. Clin Chem.

[19] Grundy SM, Cleeman JI, Daniels SR, Donato KA, Eckel RH, Franklin BA (2005). Diagnosis and management of the metabolic syndrome: an American Heart Association/National Heart, Lung, and Blood Institute Scientific Statement. Circulation.

[20] World Health Organization (2000). Regional Office for the Western P. The Asia-Pacific perspective: redefining obesity and its treatment.

[21] Consultation WHOE (2004). Appropriate body-mass index for Asian populations and its implications for policy and intervention strategies. Lancet.

[22] Chu NF (2005). Prevalence of obesity in Taiwan. Obes Rev.

[23] Hwang LC, Bai CH, Chen CJ (2006). Prevalence of obesity and metabolic syndrome in Taiwan. J Formos Med Assoc.

[24] Chuang SY, Chen CH, Chou P (2004). Prevalence of metabolic syndrome in a large health check-up population in Taiwan. J Chin Med Assoc.

[25] Zhang D, Maalouf NM, Adams-Huet B, Moe OW, Sakhaee K (2014). Effects of sex and postmenopausal estrogen use on serum phosphorus levels: a cross-sectional study of the National Health and Nutrition Examination Survey (NHANES) 2003-2006. Am J Kidney Dis.

[26] Koek WNH, Campos-Obando N, van der Eerden BCJ, Rijke YB de, Ikram MA, Uitterlinden AG (2021). Age-dependent sex differences in calcium and phosphate homeostasis. Endocr Connect.

[27] Aoki K, Miyagawa K (1990). Correlation of increased serum calcium with elevated blood pressure and vascular resistance during calcium infusion in normotensive man. J Hypertens.

[28] Sabanayagam C, Shankar A (2011). Serum calcium levels and hypertension among U.S. adults. Clin Hypertens (Greenwich).

[29] Hunt SC, Williams RR, Kuida H (1991). Different plasma ionized calcium correlations with blood pressure in high and low renin normotensive adults in Utah. Am J Hypertens.

[30] Sun G, Vasdev S, Martin GR, Gadag V, Zhang H (2005). Altered calcium homeostasis is correlated with abnormalities of fasting serum glucose, insulin resistance, and beta-cell function in the Newfoundland population. Diabetes.

[31] Hagström E, Hellman P, Lundgren E, Lind L, Arnlöv J (2007). Serum calcium is independently associated with insulin sensitivity measured with euglycaemic-hyperinsulinaemic clamp in a community-based cohort. Diabetologia.

[32] Ahlström T, Hagström E, Larsson A, Rudberg C, Lind L, Hellman P (2009). Correlation between plasma calcium, parathyroid hormone (PTH) and the metabolic syndrome (MetS) in a community-based cohort of men and women. Clin Endocrinol (Oxf).

[33] Kim MK, Kim G, Jang EH, Kwon HS, Baek KH, Oh KW (2010). Altered calcium homeostasis is correlated with the presence of metabolic syndrome and diabetes in middle-aged and elderly Korean subjects: the Chungju Metabolic Disease Cohort study (CMC study). Atherosclerosis.

[34] Revankar CM, Cimino DF, Sklar LA, Arterburn JB, Prossnitz ER (2005). A transmembrane intracellular estrogen receptor mediates rapid cell signaling. Science.

[35] Li S, Li Y, Ning H, Na L, Niu Y, Wang M (2013). Calcium supplementation increases circulating cholesterol by reducing its catabolism via GPER and TRPC1-dependent pathway in estrogen deficient women. Int J Cardiol.

[36] Cifuentes M, Fuentes C, Mattar P, Tobar N, Hugo E, Ben-Jonathan N (2010). Obesity-associated proinflammatory cytokines increase calcium sensing receptor (CaSR) protein expression in primary human adipocytes and LS14 human adipose cell line. Arch Biochem Biophys.

[37] Vaidya A, Underwood PC, Annes JP, Sun B, Williams GH, Forman JP (2013). The influence of sodium- and calcium-regulatory hormone interventions on adipocytokines in obesity and diabetes. Metabolism.

[38] Reis JP, von Mühlen D, Miller ER (2008). Relation of 25-hydroxyvitamin D and parathyroid hormone levels with metabolic syndrome among US adults. Eur J Endocrinol.

